# An echocardiographic study of infective endocarditis, with special reference to patients with HIV

**DOI:** 10.5830/CVJA-2013-084

**Published:** 2014-04

**Authors:** SH Nel, DP Naidoo

**Affiliations:** Department of Cardiology, University of KwaZulu-Natal, Durban, South Africa; Department of Cardiology, University of KwaZulu-Natal, Durban, South Africa

**Keywords:** infective endocarditis, HIV, rheumatic heart disease

## Abstract

**Objective:**

The aim was to describe the echocardiographic features of patients with infective endocarditis (IE), and to compare the manifestations of IE in HIV-positive versus HIV-negative patients.

**Methods:**

The study was prospective in nature and screened patients referred to Inkosi Albert Luthuli Hospital (IALCH) with suspected IE between 2004 and 2007. Only patients with a definite diagnosis of IE according to the modified Duke criteria were enrolled for the purpose of the study. Inkosi Albert Luthuli hospital is an 842-bed tertiary referral centre, serving a KwaZulu-Natal population of 10 million people, who are of various races.

**Results:**

During this period, 91 patients were screened for IE. Seventy-seven (HIV infected, *n* = 17) satisfied the criteria for a definite diagnosis of IE. Blood cultures were positive in 46% of cases. The commonest organism was *S aureus*. Most patients had advanced valve disruption with heart failure and high peri-operative mortality. The clinical profile in the HIV-infected patients was similar to the that of the non-infected patients. The prevalence of echocardiographic complications (abscesses, aneurysms, perforations, fistulae and chordal ruptures) was 50.6% in the whole group. Except for the presence of leaflet aneurysms and root abscesses in four advanced (CD4 counts < 250 /mm^3^) HIV-infected cases, complications were not more frequent in the HIV-infected group.

**Conclusion:**

There was a high rate of culture-negative cases in this study, probably related to prior antibiotic usage; in this setting the modified Duke criteria have diagnostic limitations. No significant differences in the clinical presentation of infective endocarditis were noted between HIV-infected and HIV-negative patients.

## Abstract

Important developments during the last 20 years have facilitated rapid and accurate diagnosis of infective endocarditis (IE), and recent guidelines emphasise the role of early surgical treatment when complications supervene.^1^,^2^ The emergence of prosthetic valve endocarditis, catheter-related endocarditis, and an increased incidence of antibiotic resistance has led to new challenges for the physician.^3^ From a microbiological standpoint, the rise in staphylococcal infections, and the immune paresis associated with HIV infection pose further diagnostic challenges that also have important implications for management.^3^

Bacteraemia is said to be common in HIV-positive patients, due to the numerous immunological defects present in this disease.^4^ Furthermore, in the setting of HIV exposure and altered immunity, infection is not uncommonly caused by unusual organisms, such as barbonella, salmonella, and listeria.^1^ This raises the question as to whether IE presents a somewhat different clinical and echocardiographic picture in the HIV-positive patient.

It is known that the degree of immunosuppression, manifested by a reduced CD_4_ lymphocyte count, strongly correlates with the presence of echocardiographic abnormalities.^5^ Whether the immunosuppression associated with HIV may alter the clinical picture of valvular heart disease, particularly IE, is not clear. Since a decrease in CD_4_ count is thought to predispose to HIV-associated cardiac disease, this study was designed to determine the pattern of cardiac involvement in the HIV-infected subjects who develop IE.^5^,^6^

## Methods

The study prospectively screened a total of 91 patients with features of suspected IE between 2004 and 2007. The diagnosis of IE was made on clinical grounds. The modified Duke criteria were then used to classify IE as definite or probable.^7^ Only patients with a definite diagnosis of IE according to the modified Duke criteria were enrolled for the purpose of the study. Inkosi Albert Luthuli Hospital (IALCH) is an 842-bed tertiary referral centre, serving a population of 10 million people in whom rheumatic heart disease is endemic.

The study protocol was approved by the Nelson R Mandela Research Ethics Committee (H095/04). The study has been structured in accordance with the Declaration of Helsinski (2000), which deals with research involving human subjects.

All patients with suspected IE referred from peripheral hospitals to the Department of Cardiology at IALCH were assessed by clinicians who documented the clinical features of IE. Blood sampling was performed for estimation of erythrocyte sedimentation rate (ESR), C-reactive protein (CRP), serum complement and blood cultures. Urine tested for microscopic haematuria and 12-lead electrocardiograms were performed on all patients.

All study participants were tested for HIV (diagnosis of HIV was determined by an ELISA test), after adequate pre-test counselling by a qualified counsellor. If the results were positive, a CD_4_ count was done. The stage of HIV infection was assessed using both clinical features and the level of CD_4_ count. A broad description of the echocardiographic features of IE was documented for the study population as a whole. The clinical features, laboratory results and echocardiographic findings were compared in the HIV-infected and uninfected patients.

Patients with a clinical diagnosis of suspected IE had an initial examination by transthoracic echocardiography (TTE) to look for echocardiographic evidence of infection, document haemodynamic status (valvular dysfunction, ventricular dimensions and ventricular function) and to determine the presence of predisposing conditions such as congenital, valvular or degenerative heart disease. Echocardiographic evidence of valve infection was accepted when any one of the following findings was identified: vegetation, paravalvular extension with abscess formation, or rupture and fistula formation, and prosthetic valve dehiscence. Leaflet or cuspal thickening, with/without areas of calcification found at TTE was taken as evidence of underlying rheumatic heart disease.

Transoesophageal echocardiography (TEE) was performed within 24 to 48 hours of admission if paravalvular extension was suspected, or where TTE images were suboptimal, and in the case of mechanical prosthetic valves. It was performed in most patients except where patients were rushed to emergency surgery on the basis of the transthoracic echo findings. Echocardiography was performed on a Sequoia C256 (Acuson, Germany) cardiac ultrasound machine, using a 5-MHz transthoracic transducer for TTE, and a 7-MHz multiplane transoesophageal probe for TEE.

## Statistical analysis

Baseline characteristics of all patients were evaluated to describe the demographics and to identify any underlying risk factors. Comparisons between HIV-infected and HIV-negative patients for categorical outcomes (e.g. valvular assessment) were evaluated by means of chi-square tests or Fischer’s exact tests. Where the outcome was numerical (e.g. CD4 count), Mann–Whitney tests were used to compare mean ranks in the HIV-positive and HIV-negative groups. The Student’s t-test was used to determine differences between samples. The significance level was taken at *p* < 0.05.

## Results

Of the 91 patients screened for suspected endocarditis, 63 satisfied the diagnosis of definite IE by the Duke and 78 by the modified Duke criteria. According to the modified Duke criteria, 77 patients were classified as definite IE (HIV infected, *n* = 17, uninfected, *n* = 60), nine as possible IE, and five as rejected IE. The analysis that follows was performed on the 77 patients with definite IE (Table 1).

**Table 1:** Duke criteria versus modified Duke criteria in the classifications of infective endocarditis

	*Duke criteria*			*Modified Duke criteria*		
	*HIV+ n = 18 (%)*	*HIV– n = 73 (%)*	*Total 91*	*HIV+ n = 18 (%)*	*HIV– n = 73 (%)*	*Total 91*
Definite	16 (88.9)	47 (64.4)	63	17 (94.4)	60 (82.2)	77
Possible	2 (11.1)	21 (28.8)	23	1 (5.6)	8 (10.9)	9
Rejected	0 (0)	5 (6.8)	5	0 (0)	5 (6.8)	5

The mean participant age in the whole group was approximately 30 years, with a slight male preponderance (55%). Overall, there was a slight male predominance for the occurrence of IE in both groups of patients: 55% (*n* = 43) were male and 45.5% (*n* = 35) were female (Table 2). There were no significant differences in age, admission weight (61 vs 59 kg), and temperature (36.5 vs 37°C) between the HIV-infected and uninfected groups. Fever above 38°C was noted in four HIV-infected patients. The source of infection was not apparent in most patients, nor was there any information from the history about a childhood history of rheumatic fever or valvular heart disease.

**Table 2:** Demographic data and clinical features in HIV-positive and HIV-negative patients with infective endocarditis

*Parameter*	*HIV+ n = 17 (%)*	*HIV– n = 60 (%)*	*Total n = 78 (%)*	p*-value*
Age (years)	32 (22–50)*	31(12–64)*	63(80.8)	0.867
Gender: male	9 (53)	33 (55)	43(55.1)	
female	8 (47)	27 (45)	35( 44.9)	
Body weight (kg)	61 (41–82)*	59 (43–79)*	120 (153.8)	0.585
Fever	4 (23.5)	3 (5)	7 (9.1)	0.024
Clubbing	11 (64.7)	32 (53.3)	43 (55.8)	0.102
Splinter haemorrhages	2 (11.8)	3 (5)	5 (6.5)	0.304
Emboli/stroke	3 (17.6)	6 (10)	9 (11.7)	1.000
Splenomegaly	2 (11.8)	3 (5)	5 (6.5)	
Heart failure/hepatomegaly	5 (29.4)	28 (47)	33 (43)	0.204
Haematuria	3 (17.6)	19 (31.7)	22 (28)	

*Mean values with the ranges bracketed.

Other clinical features of infective endocarditis did not appear to be different in the two groups. Among the 17 HIV-infected patients, 11 had clubbing and five had heart failure. Hepatomegaly mirrored the findings of congestive heart failure and was found in five (29.4%) HIV-infected patients, and in 28 (46.7%) HIV-negative patients (*p* = 0.024) (Table 2).

Thirty-six of the 78 patients with definite IE (46%) had positive blood cultures; 29 (37%) of these were from the HIV-negative patients, and seven (9%) were from the HIV-infected patients. *S aureus* was the commonest infecting bacterium in both groups of patients, and was found overall in 17 (47%) of those with positive cultures. Of these, four were HIV-infected and 13 were HIV negative (*p* = ns). The second most common infecting bacterium was *S viridans* (*n* = 7) (20%); of these one was in the HIV-infected group. One HIV-infected patient had an unusual organism; this was *Propionibacterium*.

Significantly, higher elevation in the sedimentation rate and the C-reactive protein levels was noted in the HIV-infected group compared to the HIV-negative group. In addition, serum albumin level was markedly lower in the HIV-infected group (Table 3).

**Table 3:** Comparison of laboratory features of HIV-positive and HIV-negative patients with infective endocarditis

*Laboratory findings*	*HIV+ n = 17 (%)*	*HIV– n = 60 (%)*	p*-value*
White blood count (/l)	7.7 (2.46–23.14)	9 (4–29.4)	0.387
Lymphocyte (/l)	2.76 (0.44–18.2)	2.93 (0.26–6)	0.548
Platelets (/l)	273 (123–449)	229 (40–432)	0.675
Haemoglobin (mg/dl)	8.92 (5–11.2)	10.76 (6–14.2)	0.119
Sedimentation rate (mm/h)	110.8 (65–142)	62.5 (6–160)	0.024
C-reactive protein (mg/dl)	95.19 (0.17–265.3)	68 (0.02–336.4)	0.018
Urea (mmol/l)	7.6 (3–192)	13.87 (1.4–28.3)	0.091
Creatinine (mmol/l)	131.6 (57–770)	201.42 (43–851)	0.301
Serum albumin (g/dl)	26.94 (18–36)	33.6 (0.57–49)	0.031
Complement C3 (g/l)	1.48 (1.1–1.77)	1.09 (0.15–1.8)	0.001
Complement C4 (g/l)	0.308 (0.13–0.46)	0.25 ( 0.01–0.52)	0.120
Rheumatoid factor (+)	4 (23.6)	33 (55)	0.052
Haematuria	3 (17.6)	19 (31.7)	

Mean values with the ranges bracketed, except rheumatoid factor and haematuria.

## Echocardiographic findings

All but one patient had findings suggestive of IE on TTE. These included the presence of vegetations, root abscesses or leaflet aneurysms (Table 4). Apart from these changes, 60 patients (78%) had leaflet thickening with/without reduced mobility or calcium on the valve apparatus that was suggestive of rheumatic heart disease; of these 51 were HIV negative, and nine were HIV infected. The remaining 17 patients had normal valves (nine HIV negative and eight HIV infected). Congenital defects were found in five patients [patent ductus arteriosus (one), bicuspid aortic valve (one), and ventricular septal defect (three) (one HIV infected and two HIV negative).

**Table 4:** Echocardiographic findings in HIV-positive and HIV-negative patients with infective endocarditis

	*HIV+ n = 17 (%)*	*HIV– n = 60 (%)*	*Total n = 77 (%)*	p*-value*
Vegetations	11 (64.7)	57 (95)	68 (88.3)	0.447
Leaflet aneurysm	4 (23.5)	1 (1.7)	5 (6.5)	0.008
Abscess	3 (17.6)	3 (5)	6 (7.8)	0.118
Regurgitation	16 (94.1)	59 (98.3)	75 (97.4)	
Pericardial effusion	6 (35.3)	26 (43.3)	28 (36.4)	1.000
Chordal rupture/leaflet prolapse	6 (35.3)	20 (33.3)	26 (33.8)	

Fifty-four (70.1%) patients underwent TEE. Of these, 51 had features suggesting IE. The remaining three patients with negative findings on TEE had thickened calcified valves (one), thickened leaflets with chordal rupture (one), and the last one had a normal valve.

**Fig. 1.**
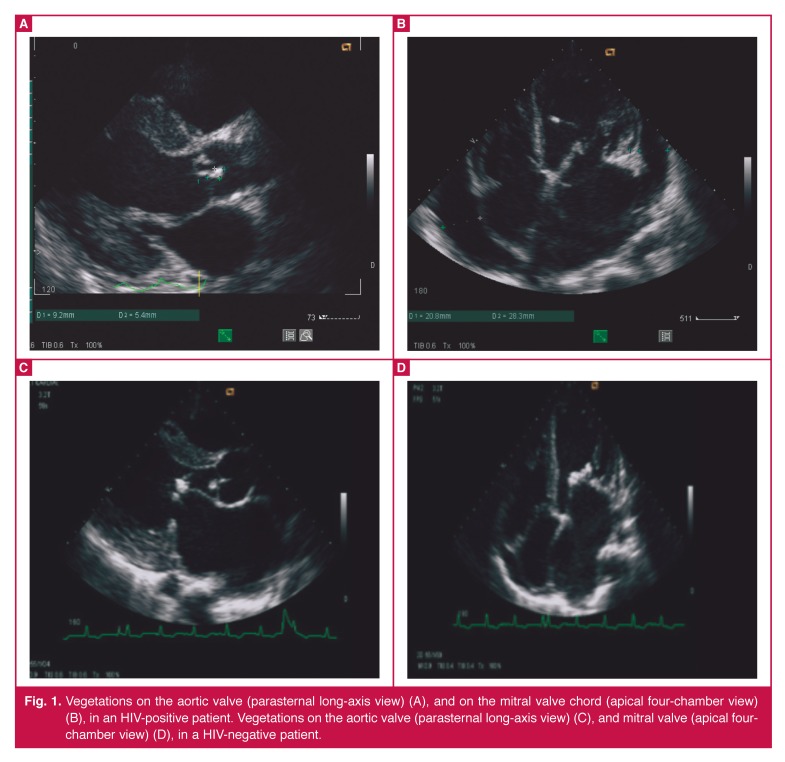
Vegetations on the aortic valve (parasternal long-axis view) (A), and on the mitral valve chord (apical four-chamber view) (B), in an HIV-positive patient. Vegetations on the aortic valve (parasternal long-axis view) (C), and mitral valve (apical fourchamber view) (D), in a HIV-negative patient.

**Fig. 2.**
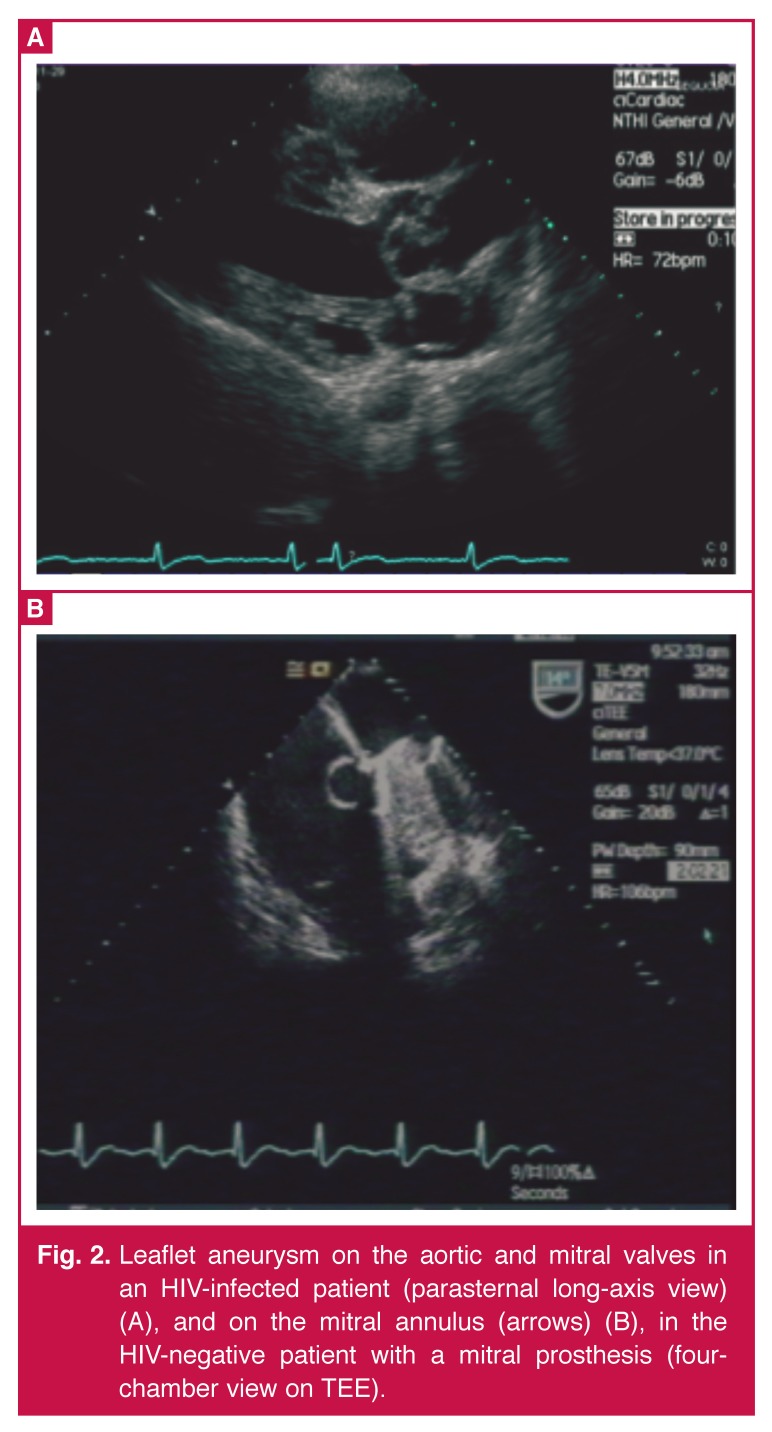
Leaflet aneurysm on the aortic and mitral valves in an HIV-infected patient (parasternal long-axis view) (A), and on the mitral annulus (arrows) (B), in the HIV-negative patient with a mitral prosthesis (four-chamber view on TEE).

**Fig. 3.**
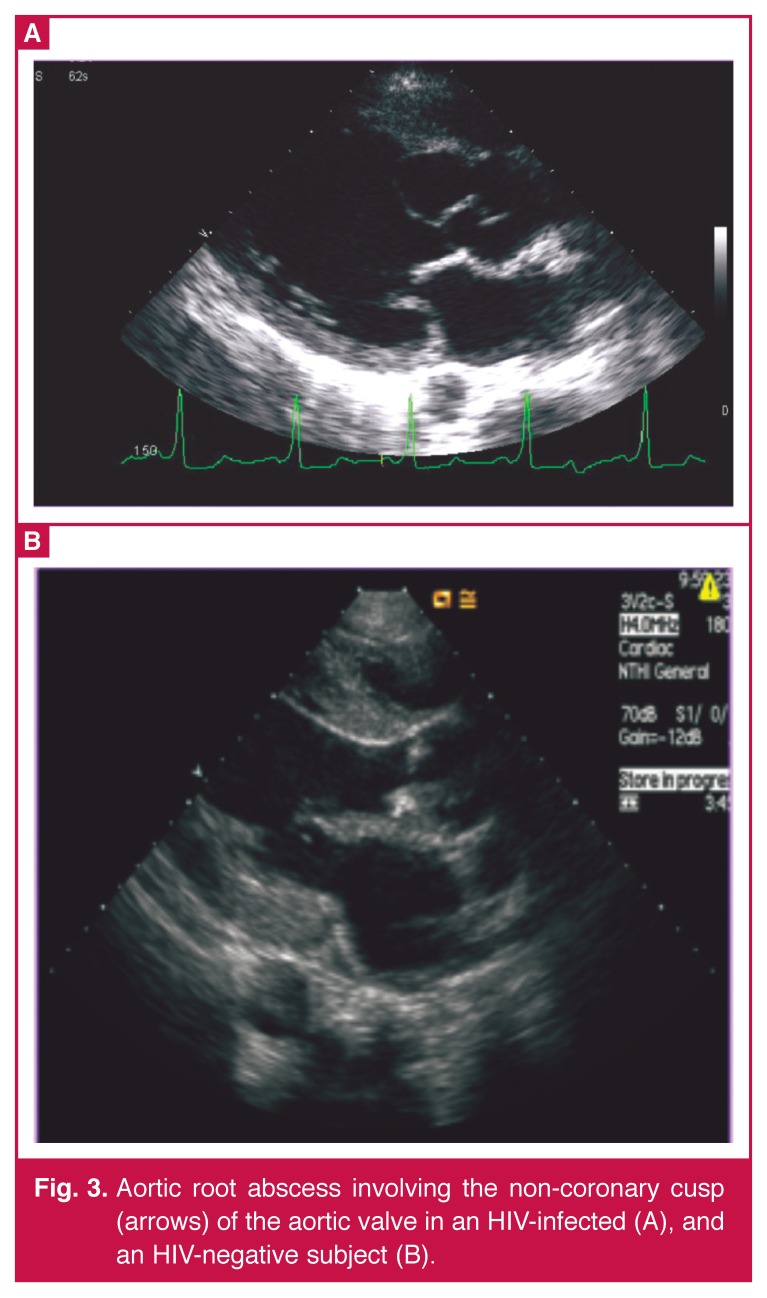
Aortic root abscess involving the non-coronary cusp (arrows) of the aortic valve in an HIV-infected (A), and an HIV-negative subject (B).

Vegetations were the predominant finding in 68 (88%) patients at echocardiography (Table 5). They were present in 11/17 (64.7%) of the HIV-infected patients, and in 57/61 (95%) of the HIV-uninfected patients. The remaining nine patients (six HIV-infected and three HIV-uninfected patients) did not have vegetations, but did show other echocardiographic features suggestive of IE. These were leaflet aneurysms in four and aortic root abscesses in four cases; one patient had a disrupted aortic valve without the presence of vegetations (Figs 1, 2–3).

**Table 5:** Vegetation characteristics in HIV-positive and HIV-negative subjects

	*HIV+ n = 17*	*HIV– n = 60*	*Total*	p*-value*
Site				
Aortic	2 (11.8)	21 (35)	23 (29.9)	0.189
Mitral	4 (23.6)	21 (35)	25 (32.5)	0.001
Tricuspid	2 (11.8)	1 (1.7)	3 (3.9)	
Other site***	0 (0)	4 (6.7)	4 (5.2)	
Mixed (aortic + mitral)	3 (17.6)	10 (16.7)**	13 (16.9)	
Mean size (mm)	11 (4–24)*	10 (3–30)*	10 (3–30)*	0.447
Vegetation number				
Single	6 (35.3)	32 (51.7)	38 (49.4)	
Multiple	5 (29.4)	25 (41.7)	30 (38.9)	
Total, *n* (%)	11 (64.7)	57 (95)	68 (88.3)	

Values expressed in brackets indicate percentages. *Mean values with the ranges bracketed **Includes one patient with a VSD who had mitral and tricuspid valve vegetations ***Other site refers to central line, pulmonary and prosthesis valves. The left atrial mural endocarditis is included with the mitral valve.

In comparison with adjacent valvular tissue, vegetations appeared echogenic or homogenous in appearance, with an irregular shape. Single and multiple vegetations were seen with the same frequency in each group. Vegetation size was similar (11 mm) in each group (*p* = ns). There was no relationship between the size of the vegetation and the presence of complications such as abscess formation, aneurysm, stroke or fistula development.

**Fig. 4.**
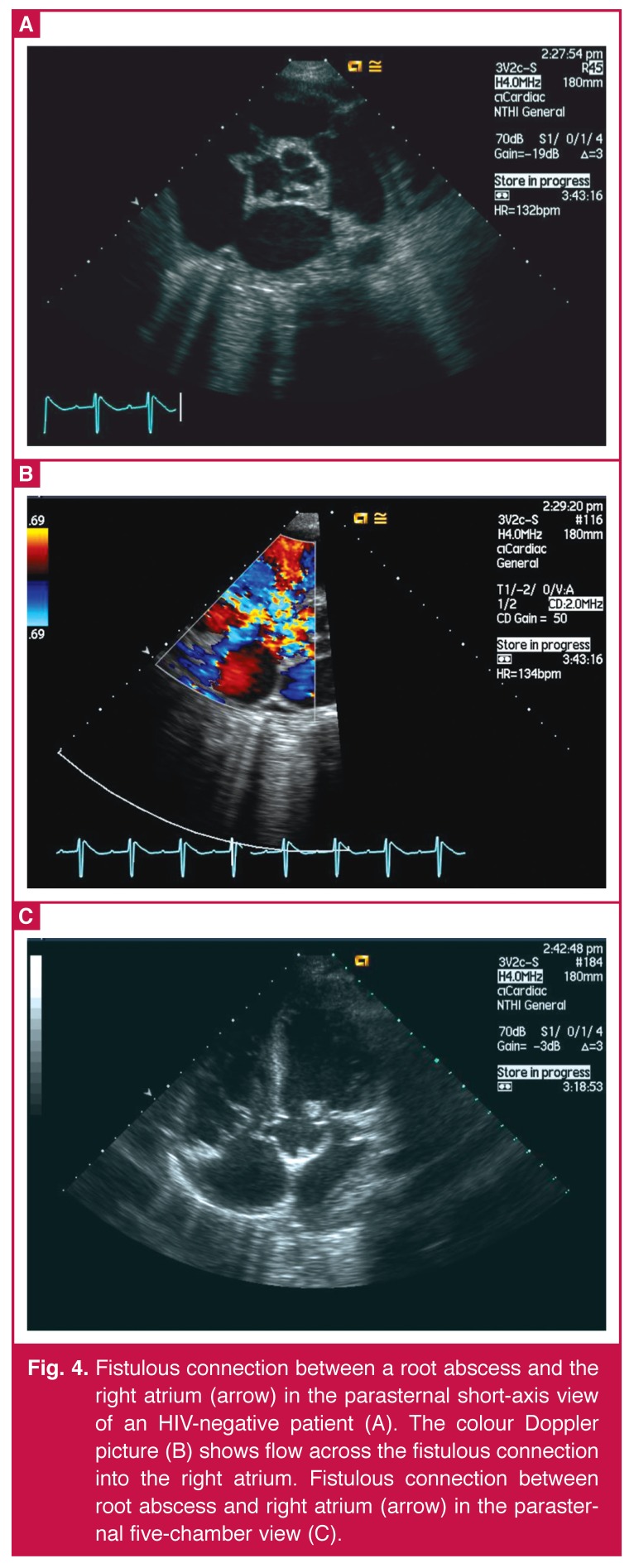
Fistulous connection between a root abscess and the right atrium (arrow) in the parasternal short-axis view of an HIV-negative patient (A). The colour Doppler picture (B) shows flow across the fistulous connection into the right atrium. Fistulous connection between root abscess and right atrium (arrow) in the parasternal five-chamber view (C).

Complications occurred in four of the 13 HIV-negative patients with vegetations larger than 10 mm. These were aortic root abscess (one), fistula (one) (Fig. 4), and two had strokes. None of the four HIV-infected patients with vegetations > 10 mm (11, 11, 11 and 11.6 mm) had complications associated with large vegetations (*p* = ns) .

Extensive valve disruption was present in both groups, since patients presented at an advanced stage of infection. Except for the leaflet aneurysms and root abscesses, which were present in four and three HIV-infected patients, respectively, (two had both aneurysm and abscess, one had an abscess, one had an aneurysm), there did not appear to be any difference in the prevalence of valve-related complications between the two groups. Of the five aneurysms in the study, two were cuspal aneurysms of the aortic valve, (both HIV infected) and the remaining three aneurysms were located on the mitral valve (two HIV infected).

Therefore, among the HIV-infected patients, leaflet aneurysms were found in the mitral (two) and aortic (two) position, and one HIV-negative patient had a leaflet aneurysm associated with a vegetation < 10 mm, affecting the mitral valve. One leaflet aneurysm was found along the annulus of a mitral prosthesis in an HIV-negative patient, confirmed at TEE (Fig. 2b)

Aortic root abscesses were present in three patients in each group, and were of a larger size in the HIV-infected (0.73 × 1.2 cm) than the HIV-uninfected patients (0.3 × 0.45 cm), but this difference was not significant (*p* = 0.118) and was not related to a very low CD4 count in two patients (15, 150, 249 /mm^3^). There was no evidence of myocardial abscess formation. Like the aortic root abscesses, the leaflet aneurysms were larger in the HIV-infected patients (0.86 × 0.85 cm vs 0.21 × 0.3 cm) (*p* = 0.008).

The mean ejection fraction (EF) in the HIV-uninfected patients was 59%, and in the HIV-infected patients, 62.9% (*p* = ns). Pericardial effusion was common and associated with heart failure and fluid overload in both groups *p* = ns). Twenty-two of the 26 HIV-uninfected patients with pericardial effusions had severe valvular regurgitation; 12 (46.2%) showed signs of heart failure with fluid overload, and two of the remaining 10 patients had impaired systolic function (EF = 35% in both). All six HIV-infected patients with pericardial effusion had severe valvular regurgitation, two with heart failure and fluid overload, and none had impaired systolic function.

## HIV stage and echocardiographic features

The mean CD_4_ count in the HIV-infected patients was 189 /mm^3^. To determine any association with the stage of immunodeficiency, the echocardiographic findings were examined in the HIV-infected patients and stratified into two groups: CD4 counts < 200 /mm^3^ and > 200 /mm^3^. No striking differences emerged between the groups in vegetation size and number of valves affected, complication rate, organism, ejection fraction or outcome.

Three of the four patients with leaflet aneurysms, and all of those with aortic root abscess had CD_4_ counts < 250 /mm^3^. The four patients with S aureus infection all had CD_4_ counts < 250 /mm^3^ (248, 231, 149 and 139 /mm^3^). Three of the four patients with very low CD_4_ counts (< 100 /mm^3^) had vegetations, and the fourth had an aortic root abscess without vegetations.

## Surgical findings

In all patients, medical therapy with appropriate antibiotics had been instituted and continued for a total period of six weeks. Forty patients (34 HIV uninfected and six HIV infected) underwent valve-replacement surgery. At surgery, the underlying valve pathology was considered to be rheumatic in origin in 38 cases (95%). In the two remaining cases, the underlying valve was considered normal by the operating surgeon. Among the six HIV-infected cases, surgery revealed underlying rheumatic valve pathology in five patients; in the remaining patient the morphology of the underlying valve tissue was normal.

Eighteen (23.4%) patients (14 HIV uninfected and four HIV infected) died during the course of the study (*p* = ns). Three HIV-uninfected patients died after surgery. One had an aortic root abscess with fistula formation with coronary ostial occlusion and died in theatre. The second died on day seven in cardiogenic shock, and the third on day eight from an intracerebral haemorrhage.

The CD_4_ counts in the HIV-infected patients who died were 139, 135, 149 and 249/mm^3^. One of these patients underwent emergency surgery for a disrupted right coronary cusp and died 35 days after surgery from a methicillin-resistant staphylococcal (MRSA) septicaemia acquired postoperatively. The remaining five HIV-infected patients who had surgery have not shown any features of re-infection, and remained stable after one year.

Six patients (7.8%), all HIV negative, had advanced renal involvement. Four were receiving haemodialysis at the time of diagnosis.

## Discussion

Few data exist on the clinical profile and echocardiographic findings of IE in HIV-infected patients in the developing world.^8^,^9^ Most reports of IE in HIV-infected individuals have focused almost exclusively on IE in intravenous drug users, and it has reportedly been rare in non-drug users.^10^ In this study we have shown that the clinical profile of IE in the HIV-positive patient is similar to that in the HIV-negative patient, and is characterised by fever, clubbing, murmurs and severe valve regurgitation. In contrast to Western series,^10^ the most common underlying predisposing abnormality observed in our study was rheumatic heart disease.

Vegetations occurred on the mitral and aortic valves and there were three cases of right-sided endocarditis in patients with congenital heart disease (two of whom were HIV infected. The mean size of the vegetations was similar in both groups (11 mm) (Table 6); three out of the four patients with CD_4_ counts < 200/mm^3^ had slightly larger-sized vegetations (13 mm). These findings are in keeping with the report by Smith *et al.* who documented an 11.5% prevalence of infective endocarditis in HIV-positive subjects with bacteraemia.11 These authors showed that there was no difference in the clinical characteristics of HIV-positive patients with and without IE.^11^

**Table 6:** Echocardiographic features predictive of surgery

*Echo finding*	*HIV+ n = 17(%)*	*HIV– n = 60 (%)*	*Total*
Vegetations			
Persistence after stroke	1 (5.9)	–	1
> 10 mm	4 (23.5)	13 (21.7)	17
Increase in size	–	1 (1.7)	1
Valve dysfunction			
Perforated leaflets	1 (5.9)	1 (1.7)	2
Valve regurgitation	16 (94.1)	55 (91.7)	71
Impaired LV function	–	3 (5)	3
Not responding to antibiotics	–	1 (1.7)	1
Paravalvular extension			
Rupture/fistulae	–	1 (1.7)	1
Abscess/aneurysm	7 (41.2)	4 (6.7)	11

We noted that leaflet aneurysms and aortic root abscesses occurred in both HIV-positive and HIV-negative subjects, but the numbers were too small for a formal statistical comparison. In these cases, echocardiography revealed severe valve damage, with peri-valvular extension of the infection leading to abscess formation and/or the development of fistula, features associated with a poor prognosis.^12^ Three patients died in the immediate postoperative phase (the first, coronary ostial occlusion, the second had cardiogenic shock, and the third patient was HIV positive and died from MRSA infection).

A relationship between aneurysms and the HIV infection has been documented in vascular series,^13^ in which aneurysms have been found to be multiple and occur in unusual anatomical locations. In our cases, the root abscesses and leaflet aneurysms were larger in size in the HIV-positive patients. None of our patients had evidence of mycotic aneurysms elsewhere, which has been reported more frequently when presentation is delayed, especially in developing countries.^14^

In this study, pericardial effusion was a common finding in both groups, and was attributed largely to severe valve regurgitation and resulting heart failure. More than half (62%) the patients with pericardial effusion had failure of leaflet or cuspal coaption with a marked degree of haemodynamic compromise. However, markedly impaired systolic function with heart failure was not more frequent in HIV-negative patients.

Pericardial effusions are considered to be a common form of cardiovascular involvement in HIV-infected individuals, the cause of which includes tuberculosis and pyogenic infection, particularly *S aureus*, as a result of endocarditis.^15^ The six patients with staphylococcal infection had small effusions that were not aspirated. Four patients in each group had evidence of extracardiac tuberculosis, but the cause of the pericardial effusion in these patients was thought to be valve destruction with heart failure.

In this study, *S aureus* was the most common infecting bacterium, followed by *S aureus*. In the Western Cape, Koegelenberg *et al.*^16^ found that *S aureus* is still the most common bacterium in their group of HIV-positive patients. In Western series, *S aureus* is the commonest causative organism in HIV-infected patients; it is reported largely in intravenous drug users and has a predilection for the tricuspid valve. None of the patients recruited in our study were intravenous (‘mainline’) drug users. We did not find any multi-resistant organisms in the HIV-infected group. In the one HIV-infected patient with MRSA, *S aureus* was acquired postoperatively.

In our study, 42 patients had negative blood cultures, an occurrence that was likely due to the setting of our study, a tertiary referral centre receiving patients who have already been started on antibiotics. In febrile patients with elevated ESR, clinicians at base hospitals (often far removed from laboratory facilities) feel obliged to administer antibiotic therapy prior to obtaining the results of initial investigations.16

A deficiency in the modified Duke criteria becomes apparent when diagnosing IE when blood cultures are negative; we have shown that these patients often have elevated sedimentation rates and C-reactive protein levels from repeated non-cardiac infection and anaemia. Using the surgical findings on operated cases as a gold standard, the positive predictive value of the modified Duke criteria was only 72%.

The higher culture negativity in HIV-infected patients in our study also raises the possibility of non-bacterial thrombotic endocarditis (NBTE) in at least some of these cases.^17^ Serial negative blood cultures should alert the clinician to the possibility of NBTE, which has been reported in HIV-infected patients.^16^ These findings have significant implications for the prevalence and diagnosis of IE in HIV-positive patients.

We found a similar rate of morbidity and mortality between HIV-infected and HIV-negative patients, in keeping with the data from Fowler *et al.*, who found that overall morbidity and mortality related to cardiac disease in AIDS was low.^18^ There were four deaths among our HIV-infected patients (23.6%), and 14 among the HIV-negative patients (23.3%). Three of the four HIV-infected patients who died had CD4 counts < 100 /mm^3^. This is in keeping with data from our centre showing that surgery in HIV-infected patients with CD4 counts of > 400 /mm^3^. are likely to have early surgical outcomes similar to that in HIV-uninfected patients.^19^

## Study limitations

The small sample size of the study was a limiting factor. This could have been due to the poor referral system from the base hospital to our hospital, or in fact, that IE is noHIV-positive patients as we had presumed. Also not all patients diagnosed with IE at TTE received a TEE. This was due to various reasons, such as a patient’s inability to tolerate the TEE probe and markedly elevated international normalised ratio (INR) levels at the time of examination.

A further limitation in the study was the high rate of negative blood cultures. This was most likely the result of administration of antibiotics to the patient prior to referral to our institution, or in the case of the HIV-positive patients, the possibility of NBTE.

Of the 91 patients initially screened, 77 were accepted as having had a definite diagnosis of IE, according to the modified Duke criteria. The remaining 14 were deemed not to have IE and excluded from the analysis. Whether any patients in this group had IE or not (true negative and false negative) could not be determined with certainty since they were not subjected to surgery.

We believe the modified Duke criteria were responsible for the higher false-positive rates since it permits diagnosis of IE based on the echocardiographic criteria in the absence of positive blood cultures. While this reflected a potential flaw in the study, since the diagnosis of IE was based on the finding of vegetations in the absence of positive blood cultures, this study highlights the difficulty in diagnosis when the blood cultures are negative, placing more reliance on echocardiographic detection of vegetations.

Unless supported by clinical features and bacteriological evidence, vegetations alone are not diagnostic of IE because they may represent healed infection. Furthermore, the diagnosis of IE postoperatively is rendered more difficult by the now common practice of leaving the chordal mechanisms intact. Five of the 33 patients clinically diagnosed as definite IE, and one possible IE, had no evidence of infection at operation, supporting the need for bacteriological confirmation of infection.

Not all patients however were referred for surgery. The low CD_4_ counts in the HIV-positive patients meant an even smaller group of these patients were accepted for operation, as the acceptable CD_4_ level for surgery at our institution is CD_4_ count > 200 /mm^3^

In this study, attempts were made to more accurately define valve pathology on echocardiography. According to Taams *et al.*, there are five distinct pathological features of IE that may be seen clearly on TEE and these are (1) mitral stenosis with vegetations; (2) myxomatous degeneration of leaflets with vegetations; (3) chordal rupture with vegetations; (4) chordal rupture without vegetations; and (5) mycotic aneurysms with fistulous connections.^20^ It is often difficult to decide on the underlying pathology with this degree of accuracy with TTE.

In our study, harmonic imaging was employed to improve the diagnostic value of TTE by improving the image quality, as documented by Chirillo *et al.*^21^ Harmonic imaging works on the principle of limiting near-field artefacts, and because the harmonic energy increases with the distance the ultrasound wave propagates, most harmonics will result from the central ultrasound beam rather than the weaker side lobe artefacts.^20^

This modality is used primarily to enhance left ventricular endocardial borders. Its use did not really increase the resolution in visualising vegetations. Therefore, we used TEE to differentiate and define chordal rupture in association with vegetations, leaflet prolapse and flail leaflets. Our surgical findings revealed that even with TEE there were limitations, which were resolved at surgery when the subtlety of the findings could not be dissected.

## Conclusion

This study has shown no significant differences in the vegetation characteristics between HIV-infected and uninfected patients. Complications such as leaflet aneurysms and root abscesses occurred in patients with CD_4_ counts < 250 /mm^3^ in HIV-positive subjects, and appeared to be of a larger size when compared to the HIV-uninfected patients. The clinical outcome of medical and surgical therapy was also similar in both groups.
